# Development and validation of a complex intervention: A physical exercise programme aimed at delaying the functional decline in frail older adults

**DOI:** 10.1002/nop2.388

**Published:** 2019-09-30

**Authors:** Rita Santos‐Rocha, Joana Freitas, Fátima Ramalho, Nuno Pimenta, Filipa Costa Couto, João Apóstolo

**Affiliations:** ^1^ Sport Sciences School of Rio Maior (ESDRM) Polytechnic Institute of Santarém (IPSantarém) Rio Maior Portugal; ^2^ Biomechanics and Functional Morphology Laboratory Faculty of Human Kinetics Interdisciplinary Centre for the Study of Human Performance University of Lisbon Cruz‐Quebrada Portugal; ^3^ Exercise and Health Laboratory Faculty of Human Kinetics Interdisciplinary Centre for the Study of Human Performance University of Lisbon Cruz‐Quebrada Portugal; ^4^ The Health Sciences Research Unit: Nursing Nursing School of Coimbra (ESEnfC), collaborator of the Joanna Briggs Institute (JBI) Coimbra Portugal

**Keywords:** complex intervention, elderly, frailty, nursing, physical exercise

## Abstract

**Aim:**

To develop and validate a physical exercise programme aimed at delaying the functional decline in frail older adults.

**Design:**

The revised guideline of Criteria for Reporting the Development and Evaluation of Complex Interventions in health care was followed.

**Methods:**

The physical exercise programme was designed and validated by exercise specialists to be implemented by healthcare professionals. The physical exercise programme underwent three stages of development, piloting and evaluation. It includes a portfolio of exercises in different support materials (posters, e‐book and website). A testing intervention was delivered to the target population.

**Results:**

The Criteria for Reporting the Development and Evaluation of Complex Interventions in health care process has the potential to help practitioners in developing and planning complex interventions, such as an exercise programme. Its components can be adjusted to the context and to the characteristics of the target population. A study protocol and a pilot study will be developed to test the effectiveness of the physical exercise programme on delaying the functional decline of frail older adults.

## 1 INTRODUCTION

The ageing process leads to the increasing demand of locomotor tasks aimed at maintaining autonomy and independence in daily life. Frailty is a prevalent geriatric syndrome observed in institutionalized older people. The lack of autonomy and the potential of falling may have significant public health implications due to the negative impact on quality of life of older adults. Maintaining functional status is an important part of an active ageing process, since it facilitates independent living, allows to reduce age‐related morbidity, improves the quality of life and reduces healthcare costs (Chodzko‐Zajko et al., 2009; Nelson et al., 2007; Skelton & Beyer, 2003).

*Physical activity* is defined as any bodily movement produced by the contraction of skeletal muscles that results in a substantial increase in caloric requirements over resting energy expenditure (Caspersen, Powell, & Christenson, 1985; American College of Sports Medicine [ACSM], 2018). *Exercise* is a type of physical activity consisting of planned, structured and repetitive bodily movement performed to improve and/or maintain one or more components of physical fitness (Caspersen et al., 1985) and/or prolong life (Boome, 2016). In other words, exercise is a subcategory of physical activity.

Exercise promotes an improvement in the physical fitness of people of all ages and health conditions. Physical activity and functional fitness play an essential role in an active and autonomous lifestyle of older adults. Moreover, exercise is considered to be the most effective strategy to treat, prevent and delay frailty (Jadczak, Dollard, Mahajan, & Visvanathan, 2018).

A systematic review concluded that physical activity reduces the age‐related decline in functional capacity and maintains muscle strength and mass among adults aged 65–85 years (Paterson & Warburton, 2010). A 50% reduction in the relative risk of developing functional limitations or disability was reported among those participating in a moderate‐intensity physical activity (Paterson & Warburton, 2010; Tak, Kuiper, Chorus, & Hopman‐Rock, 2013).

Moreover, recent systematic reviews and meta‐analysis showed strong evidence of the effectiveness of exercise in the prevention of falls and fractures in the older population (Chang et al., 2004; Gillespie et al., 2003; Shekelle et al., 2003; Sherrington, Tiedemann, Fairhall, Close, & Lord, 2011) further state that exercise can be considered the best approach to fall prevention at a population level and that up to 42% of falls can be prevented by well‐designed exercise programmes. These findings may be highly relevant because, in addition to the many positive health effects of exercise, the consequent reduction in fractures and other injuries associated with falls ought to also have a beneficial impact on healthcare costs (Rose & Hernandez, 2010; Shumway‐Cook et al., 2009).

### 1.1 Background

Encouraging frail older adults to take up a specific exercise programme is crucial in the management of the frailty condition (Jadczak et al., 2018). However, research shows that physical activity and exercise interventions lack homogeneous methods of development, delivering and assessment due to its complexity. On the other hand, the absence of structured exercise intervention models for frail older adults may be one of the obstacles to understanding the effectiveness of such programmes. Moreover, there is no structured guidance at the national level regarding the implementation of specific exercise interventions by healthcare professionals or/and exercise specialists.

According to the Medical Research Council, a complex intervention is described as an intervention that contains several interacting components, which can act either independently or interdependently (Craig et al., 2008). Educational, behaviour change and healthcare interventions are likely to be considered as complex interventions and require specific guidelines to be reported and assessed (Campbell et al., 2000; Craig et al., 2008; Michie, Fixsen, Grimshaw, & Eccles, 2009; Möhler, Köpke, & Meyer, 2015).

A physical exercise programme can be considered a complex intervention, since it is tailored to a specific population and setting and it is affected by several components regarding effectiveness and safety. Thus, the need to develop and validate well‐defined and replicable exercise protocols emerges to bridge the identified gap.

This study is part of a larger project “Mind&Gait” supported by PORTUGAL2020 program and approved by the ethics committee of the Health Sciences Research Unit: Nursing from the Nursing School of Coimbra. The physical exercise programme is part of a combined intervention. The protocol of the combined intervention describes the methods in detail, and it is published elsewhere (Apóstolo et al., 2019).

Regarding the potential influence on nursing education and research, the revised guideline of Criteria for Reporting the Development and Evaluation of Complex Interventions in health care (CReDECI2) is a formally consented reporting guideline aiming to improve the reporting quality of the development and evaluation stages of complex interventions in health care. Thus, a physical exercise programme aimed at delaying the functional decline in institutionalized frail older populations was described in accordance to CReDECI2, to help practitioners in developing and planning complex interventions, such as an exercise programme. Moreover, the materials produced provide structured guidance regarding the implementation of specific exercise programmes by health or/and exercise specialists working with institutionalized frail older adults over 65 years of age. The programme includes illustrations, description of exercises in the seated and standing positions, levels of complexity, critical points and variations.

## 2 THE STUDY

### 2.1 Aims

To develop and validate a physical exercise programme aimed at delaying the functional decline in institutionalized frail older adults.

### 2.2 Design

In 2000, the British Medical Research Council published a framework and guidelines for developing and accessing complex interventions (Moher et al., 2010) which were updated in 2008 (Craig et al., 2008), which can be accessed from (http://www.mrc.ac.uk/complexinterventionsguidance). In 2012, that framework and guidelines were developed and applied to the contexts of health care (Möhler, Bartoszek, Köpke, & Meyer, 2012) and recently updated by Möhler et al. (2015). The exercise programme aimed at delaying the functional decline in institutionalized frail older adults was designed by exercise specialists to be implemented by healthcare professionals. Thus, it was planned to include several exercises to promote functionality and with options of different positions (upright, upright with support and seated) and levels of frailty. Moreover, it included tutorial videos and pictures, using different support materials. The validation process was carried out in the first four months of 2018.

### 2.3 Participants

A total of 176 participants were involved in the process. Five groups of participants were involved in the validation process: (a) exercise specialists with PhD, MSc or BSc in exercise sciences and professional experience with older adults (6 in total); (b) healthcare specialists (nurses, physical therapists and occupational therapists) with PhD, MSc or BSc in health sciences and professional experience with older adults (5 in total); (c) undergraduate students in exercise and health sciences (139 in total); (d) exercise specialists dealing with the targeted population (6 in total); and (e) the frail older adults themselves (20 in total).

### 2.4 Instrument

The revised guideline of Criteria for Reporting the Development and Evaluation of Complex Interventions in health care (CReDECI 2) by Möhler et al. (2015) was followed.

The CReDECI 2 guideline comprises 13 items for the stages: development, piloting and evaluation and includes examples of real studies for each item (Möhler et al., 2015).

### 2.5 Ethical considerations

All procedures performed in studies involving human participants were in accordance with the ethical standards of the institutional and/or national research committee and with the 1964 Helsinki declaration and its later amendments or comparable ethical standards. This study is part of the study protocol that has been approved by The Ethics Committee of UICISA: E Board Affiliation: Health Sciences Research Unit: Nursing, of the Nursing School of Coimbra, with the approval number P455‐09/2017 (Apóstolo et al., 2019). All groups of participants were informed about the objectives and the nature of the study, as also possible risks and benefits and the details of their involvement. They were informed that they have the right to withdraw from the study at any time, and this would not result in any penalty or difference in their future treatment or care. All the participants gave their written informed consent prior to participation in the study. Students and professionals were invited to attend free educational workshops to present the exercise programme and to collect feedback to improve it. Frail older participants were invited to attend free exercise classes. No adverse events were reported while testing the programme in a group of frail elders. All materials produced by the research team were made available to the participating institutions.

## 3 RESULTS

**Figure 1:**
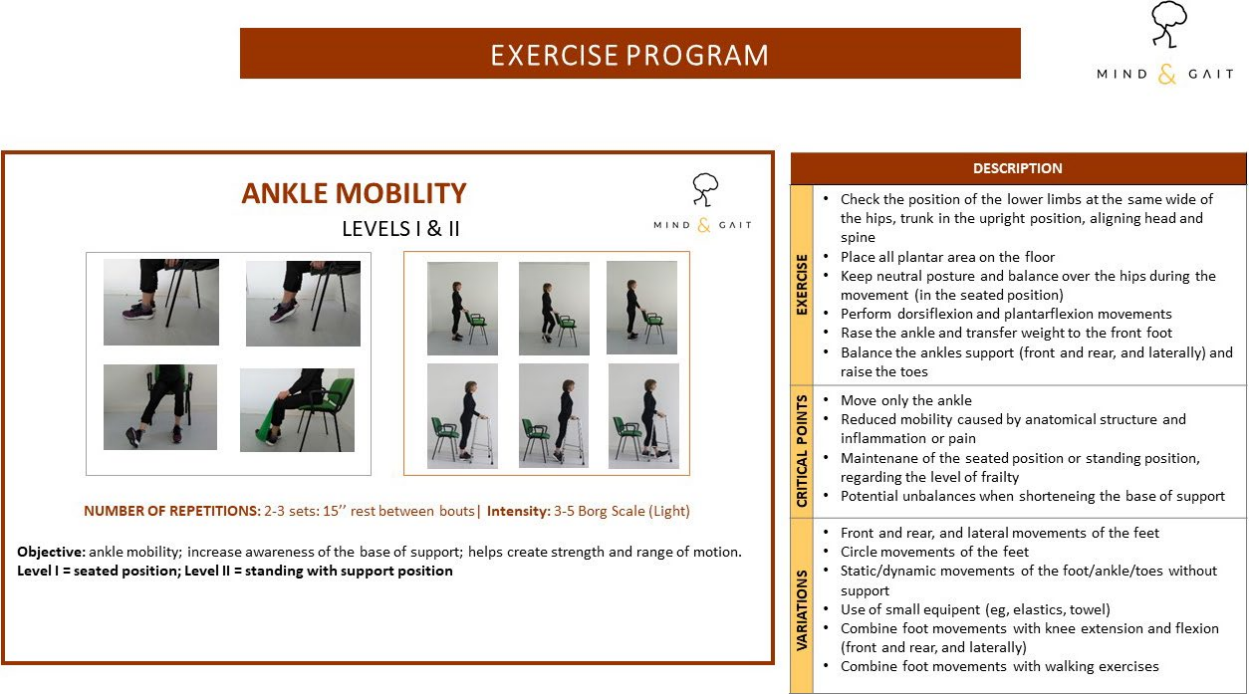
Layout of the exercise program

A portfolio of exercises including walking with and without walking aids, light resistance exercises for the lower and upper limbs, breathing and posture exercises, mobility and relaxation was developed by means of different support materials such as a slideshow presentation (Figure 1), a digital manual (e‐book), a website and an informatics application (“app”). For each exercise, few pictures, a description of the objectives, the main critical aspects, the exercise in the upright position (with and without support) and in the seated position, the options for increasing or decreasing intensity and complexity, and a tutorial video were provided. A poster showing the importance of exercise in the older populations and a poster with a short version of a periodized sequence of exercises were also developed. The exercise programme was described in the Portuguese language and published in digital format (Santos‐Rocha, Freitas, Ramalho, Couto, & Apóstolo, 2019). The exercise programme underwent the three stages proposed by Möhler et al. (2015): development, piloting and evaluation, as follows.

### 3.1 First stage: development

#### 3.1.1 Item 1—description of the intervention's underlying theoretical basis

The functional status of older adults promotes independent living (Chodzko‐Zajko et al., 2009; Nelson et al., 2007; Plummer, Zukowski, Giuliani, Hall, & Zurakowski, 2015). There is consistent literature regarding the impact of exercise programmes on functional fitness parameters, despite the heterogeneity of the interventions and the fact that most of the studies are focused on clinical populations (Chen, Lin, & Jiang, 2015; Parmenter, Raymond, Dinnen, & Singh, 2011; Rubenstein et al., 2000; Thuné‐Boyle, Iliffe, Cerga‐Pashoja, Iliffe, Cerga‐Pashoja, Lowery, & Warner, 2012; Tseng, Gau, & Lou, 2011). Among older groups (aged 70–75 years), the functional status may be preserved with even lower amounts of physical activity. The exercise prescription framework provided by ACSM (2018) was followed regarding the sequence of the exercises and the structure of each session.

#### 3.1.2 Item 2—description of all intervention components, including the reasons for their selection andtheir aims/essential functions

The exercise programme includes several components that can be adjusted to the context and to the characteristics of the target population: frail older adults with limited autonomy regarding gait. It includes a portfolio of exercises in different support materials (Santos‐Rocha et al., 2019). The exercise programme is organized in six basis sessions of 30 min, regarding two levels of difficulty and three periods of periodization: adaptation (2 weeks), improvement (6 weeks) and maintenance (4 weeks). Each session follows a conventional structure (ACSM, 2018) and addresses the health‐related fitness components. An image and a video of each exercise are provided, as well as its description, objectives, critical aspects (technique), safety and variations of intensity (options for less or more intensity and complexity). The same exercise performed in the upright position with and without support, as well as the seated position. The requirements of space and materials to be used and optional equipment are also addressed.

#### 3.1.3 Item 3—illustration of any intended interactions between different components

The exercise programme also includes a single‐structured 75‐min workshop to be provided to healthcare professionals for each cluster of the intervention group. This workshop intends to sensitize healthcare professionals about the structure of each session and the best use of the exercise programme guide. The guide will be provided by means of an e‐book, an informatics application (“app”) and a poster.

#### 3.1.4 Item 4—description and consideration of the context's characteristics in intervention modelling

The exercise programme was planned to be delivered in older end‐users organizations, to groups of up to 10 participants that need a walking aid in the upright position, or a chair in the seating position. Each participant requires at least two square metres of free space around the place in the room. The environment must be clean and comfortable regarding temperature, acoustic and safety conditions. Exercises can also be performed outdoors if the weather conditions are favourable. The healthcare professional or exercise specialist must keep visual contact with the group during the entire session and provide proper feedback. Music is optional.

### 3.2 Second stage: feasibility and piloting

#### 3.2.1 Item 5—description of the pilot test and its impact on the definite intervention

The pilot tests aimed to determine the feasibility, acceptability and practicability of the exercise programme and the supporting materials. The exercise programme and the supporting materials were tested with the five groups of participants described in the methods section, as follows.

The first version of the exercise programme was developed by three exercise specialists (JPF, FR, RSR) and was delivered in mid‐January by means of a slideshow presentation among the research team. The first version of the exercise programme in the format of a digital manual (e‐book) was delivered by the end of January and was shared among six exercise specialists (NP, SF, VS, LR, IV, FM) and four health/nursing specialists (JA, FCC, MD, JR). Moreover, a 75‐min workshop was delivered between January and February to the following stakeholders: a group of exercise professionals from Leiria, a group of sports, fitness and health students from Rio Maior, a group of healthcare professionals working in nursing homes frail elders from Rio Maior and Coimbra and three groups of nursing and physiotherapy students from Coimbra, Leiria and Santarém.

After each workshop, an anonymous questionnaire was provided to each (professional or student), to collect their feedback about the exercise programme, regarding the structure, duration, frequency, types of exercises, variations, progression, equipment, feasibility, tutorials, etc. The participants were invited to provide feedback in 12 questions using a 5‐points Likert scale, ranging from “absolutely agree” to “absolutely disagree”, except in questions 11 and 12, which included three options (i.e. “both,” “app,” “e‐book” and “both”, “printed book,” “poster,” respectively). The questionnaire also included an open question (13) and three questions about occupation, gender and age of respondents. Table 1 shows the questions asked and feedback given by the workshop’ participants.

**Table 1:** Questions asked and feedback given by the workshop’ participants

		Nursing students (*N* = 52)	Occupational therapy students (*N* = 41)	Exercise students (*N* = 40)	Exercise professionals (*N* = 6)	Total (%)
1—The exercise programme is well‐structured?	*(5) absolutely agree*	27	14	21	2	46
	*(4) agree*	21	25	17	4	48
	*(3) neither agree nor disagree*	4	1	2	0	5
	*(2) disagree*	0	0	0	0	0
	*(1) absolutely disagree*	0	0	0	0	0
2—The duration (30 min sessions) of the exercise programme is adequate?	*(5) absolutely agree*	22	17	19	0	42
	*(4) agree*	22	20	20	2	46
	*(3) neither agree nor disagree*	5	2	1	1	6
	*(2) disagree*	2	2	0	3	5
	*(1) absolutely disagree*	1	0	0	0	1
3—The frequency (three times per week) of the exercise programme is adequate?	*(5) absolutely agree*	18	15	9	1	31
	*(4) agree*	30	25	24	4	60
	*(3) neither agree nor disagree*	3	1	4	1	6
	*(2) disagree*	1	0	2	0	2
	*(1) absolutely disagree*	0	0	0	0	0
4—The variety of exercises provided in the programme is adequate to the targeted population?	*(5) absolutely agree*	29	9	15	3	40
	*(4) agree*	18	29	25	3	54
	*(3) neither agree nor disagree*	5	3	0	0	6
	*(2) disagree*	0	0	0	0	0
	*(1) absolutely disagree*	0	0	0	0	0
5—The variations of exercises provided in the programme are adequate to the targeted population?	*(5) absolutely agree*	21	8	16	3	35
	*(4) agree*	26	25	22	3	55
	*(3) neither agree nor disagree*	5	8	2	0	11
	*(2) disagree*	0	0	0	0	0
	*(1) absolutely disagree*	0	0	0	0	0
6—Is the equipment adequate to the targeted population?	*(5) absolutely agree*	23	5	20	3	37
	*(4) agree*	20	26	17	3	47
	*(3) neither agree nor disagree*	8	10	2	0	14
	*(2) disagree*	1	0	0	0	1
	*(1) absolutely disagree*	0	0	0	0	0
7—Is the structure of each session‐model adequate to the targeted population?	*(5) absolutely agree*	24	5	18	2	35
	*(4) agree*	25	33	20	3	58
	*(3) neither agree nor disagree*	3	2	2	1	6
	*(2) disagree*	0	1	0	0	1
	*(1) absolutely disagree*	0	0	0	0	0
8—Is the structure of each session‐model clear and easy to follow by the professionals?	*(5) absolutely agree*	29	13	25	2	50
	*(4) agree*	18	23	14	1	40
	*(3) neither agree nor disagree*	5	5	1	2	9
	*(2) disagree*	0	0	0	1	1
	*(1) absolutely disagree*	0	0	0	0	0
9—Are the explanations about each exercise clear to the professionals?	*(5) absolutely agree*	26	12	26	2	47
	*(4) agree*	21	21	13	2	41
	*(3) neither agree nor disagree*	3	7	1	2	9
	*(2) disagree*	2	1	0	0	2
	*(1) absolutely disagree*	0	0	0	0	0
10—Does the exercise programme have potential to be replicated (feasible in nursing homes)?	*(5) absolutely agree*	23	11	21	1	40
	*(4) agree*	23	23	14	4	46
	*(3) neither agree nor disagree*	5	6	5	1	12
	*(2) disagree*	1	1	0	0	1
	*(1) absolutely disagree*	0	0	0	0	0
11—Should the exercise programme be available to the professionals by means of an e‐book or an informatics application?	*(3) both*	13	13	6	1	24
	*(2) app*	22	15	12	4	38
	*(1) e‐book*	17	13	22	1	38
12—Should the exercise programme be available to the professionals by means of a printed book or posters?	*(3) both*	17	7	0	0	17
	*(2) printed book*	19	30	35	5	64
	*(1) posters*	16	4	5	1	19

Regarding the feedbacks provided to each question, most participants (86%–94%) agree or absolutely agree with the following aspects: (a) the exercise programme is well‐structured; (b) the duration (30 min sessions) of the exercise programme is adequate; (c) the frequency (three times per week) of the exercise programme is adequate; (d) the variety of exercises provided in the programme are adequate to the targeted population; (e) the variations of exercises provided in the programme are adequate to the targeted population; (f) the equipment is adequate to the targeted population; (g) the structure of each session‐model is adequate to the targeted population; (h) the structure of each session‐model is clear and easy to follow by the professionals; (i) the explanations about each exercise are clear to the professionals; (j) the exercise programme has potential to be replicated (feasible in nursing homes); (k) the exercise programme should be available to the professionals by means of an e‐book and an informatics application; and (l) the exercise programme should be available to the professionals by means of a printed book and posters.

Finally, the summary of in the open question “13—Would you like to provide any other comments?” is provided in the following list of topics:

- “add more weekly sessions”
- “apply in hospitals as well”
- “have in mind that medication may affect the participant coping”
- “have in mind environmental conditions”
- “have in mind the importance of motivating, explaining and demonstrating the exercises to participants”
- “include an advanced manual explaining the exercises and ways of delivering it”
- “include swimming exercises”
- “quite interesting and helpful for healthcare professionals”
- “should be linked with the rehabilitation programme/occupational therapist”
- “split the duration of the session at the beginning or as necessary”
- “the programme will also prevent social isolation and promote quality of life”
- “using videos as well”
- “well‐structured and quite interesting programme”

An exercise session was provided to two groups of frail older adults from Leiria and Rio Maior. Due to the frailty condition and age of participants, no questionnaires were applied. During the sessions, no incidents or injuries were reported. After the session, participants were asked if they enjoyed the exercise session and they would like to experience it every week. The feedbacks were all positive.

With this information, the programme was further developed (final version), regarding the addition of more exercises and variations and also tutorial videos explaining the critical aspects of the exercises. Moreover, a new section was inserted in the manual, further explaining the exercises and ways of delivering it, as well as the importance of motivating, explaining and demonstrating the exercises to participants.

Thus, the exercise programme was validated by exercise specialists, nurses, physical therapists and exercise sciences students and the targeted population. The final version of the exercise programme was delivered on March 2018.

The manual/guide of the exercise programme is available in e‐book format (available at a website), and a printed format and an informatics application (under construction) will be made available. Moreover, five infographics/posters were produced containing information related to (a) the promotion of the benefits of physical exercise included in an active ageing process; (b) the promotion of the “Mind&Gait” programme; (c) the structure of exercise session one (to be followed during the first 4–5 weeks); (d) the structure of exercise session two (to be followed during the next 6–12 weeks); (e) the structure of exercise session three (to be followed after 12 weeks); and (f) basic exercises to be performed every day.

### 3.3 Third stage: evaluation

#### 3.3.1 Item 6—description of the control condition (comparator) and reasons for the selection

An exercise programme combined with a cognitive stimulation programme will be delivered to a multi‐centre intervention group. A control group will receive standard care. The protocol of the study is published elsewhere (Apóstolo et al., 2019). The groups were randomized by institution (setting where the intervention takes place). The reason is to avoid having in the same institution participants engaged in the interventions while others not and also to avoid selection bias. It means that the participants of the control group are supposed to be included in a “waiting list” and to engage in a similar programme to be delivered post‐trial, under the responsibility of each partner institution and using the materials produced by the research team.

We hypothesized that a tailored exercise intervention for frail older adults would result in a delay in functional decline in institutionalized frail older adults when compared with standard care.

#### 3.3.2 Item 7—description of the strategy for delivering the intervention within the study context

The exercise programme was planned to be delivered in the healthcare context in older end‐user organizations. This programme should be included in the regular schedule of older people. In this pilot study, the exercise programme was delivered by exercise specialists. In the main study, the exercise programme will be delivered by exercise specialists. In the future, the exercise programme is supposed to be delivered by healthcare professionals supported by the app or e‐book and preferably, after attending a 2 hr workshop ministered by exercise specialists.

#### 3.3.3 Item 8—description of all materials or tools used delivery the intervention

The exercise programme was planned to be delivered in short space and using walking aids or chairs for support and for those with limitations who cannot exercise in the upright position. There are a few optional materials that can be used such as elastic rubber and hand free weights of 0.25–0.5 kg (e.g. a small bottle of water). Music can also be used for motivation according to the preferences of the participants.

To ensure the creation of awareness towards the intervention and provide guidance to the professional delivering the exercise session, a printed poster illustrating the structure of each session was produced. This poster is supposed to be shown in the room where the exercise sessions take place.

Moreover, another printed poster showing a few basic and simple exercises was produced, to encourage participants to perform those exercises daily. This poster is supposed to be shown in the rooms or social areas of the institution.

#### 3.3.4 Item 9—Description of the fidelity of the delivery process compared the study protocol

The exercise programme includes illustrations, describes each exercise in the seated and standing positions, levels of complexity, critical points and variations of exercises and small equipment. It also includes a plan organized in three different periods, including the session structure and the suggested exercises for each stage, having in mind the predefined objectives. Thus, it works as a predesigned intervention to be followed by the healthcare professional. This structure provides guidance to the professional delivering the intervention to adapt each exercise to the skill of each participant and avoids deviations to the protocol.

#### 3.3.5 Item 10—description of a process evaluation and its underlying theoretical basis

The process evaluation was planned to determine the results and success of the exercise programme. The selected outcome measures to evaluate the effectiveness of the exercise programme are gait speed, biomechanical parameters of gait (total plantar pressure, peak pressure and distribution of forces under the plantar area), as described in Apóstolo et al. (2019). The outcome measures will be assessed at baseline and after 12 weeks of intervention.

Gait is a basic form of locomotion and walking an easy and readily inexpensive form of exercise in older adults. However, ageing is associated with significant changes in foot characteristics which contribute to altered plantar loading patterns during gait. The lack of autonomy and the potential of falling may have significant public health implications due to the negative impact on the quality of life of older people. Gait ability and cognitive function are interrelated during both normal walking and dual‐task walking, and gait ability is thus adversely affected by cognitive impairment in both conditions (Doi et al., 2014).

According to Bridenbaugh and Kressig (2011) impairments in both gait and cognition are prevalent in older adults and thus, older adults with gait impairment have an increased risk of developing cognitive impairments. Garcia‐Pinillos, Cozar‐Barba, Munoz‐Jimenez, Soto‐Hermoso, and Latorre‐Roman (2016) analysed the association between physical and cognitive functions in older people, as well as the most appropriate physical test to assess cognitive impairment, functional capacity, comorbidity and perceived health in this population. The authors showed that gait speed is an important predictor of functional capacity (physical and cognitive function) in adults over 65 years old.

Bridenbaugh and Kressig (2011) suggest that quantitative gait analysis can provide early detection of gait and cognitive impairments as well as other health adverse outcomes (e.g. falls, fractures, hospitalization) and in the future, it will help to distinguish dementia subtypes in early stages of the diseases.

#### 3.3.6 Item 11—description of internal facilitators and barriers potentially influencing the delivery of the intervention as revealed by the process evaluation

Internal facilitators and barriers potentially influencing the delivery of the intervention are related to the institution strategy and vision of including an exercise programme into the daily routine of the inmates.

#### 3.3.7 Item 12—description of external conditions or factors occurring during the study which might have influenced the delivery of the intervention or mode of action (how it works)

External conditions or factors occurring during the study which might have influenced the delivery of the intervention or mode of action are related to the fact that the intervention was delivered by a specialist supported by the project.

#### 3.3.8 Item 13—Description of costs or required resources for the delivery of the intervention

The exercise programme is available to the professionals by means of a digital book and printed posters. A free video tutorial will also be available, on‐demand for these materials. The exercise programme was designed to be delivered in a regular room and requiring few types of equipment. It was planned and structured to be delivered by healthcare professionals that deal on a daily basis with institutionalized frail older adults. Thus, not requiring specific costs with materials and facilities, or extra costs with Human Resources. However, the authors of the exercise programme can also deliver a 3‐hr workshop to train nurse professionals on delivering or supervising the exercise sessions. If not covered by a funded project, this workshop may constitute an average cost of 200 euro. In the future, if institutions decide to externally contract an exercise or nurse professional to delivery of the intervention (assuming 5h/week), therefore, this may constitute an average cost of 200 euro/month (in accordance with 15% of national salary reference), if not covered by a funded project.

## 4 DISCUSSION

Current evidence supports the importance of engaging frail older adults in specific exercise programmes aiming at managing the frailty condition. However, physical activity and exercise interventions lack homogeneous methods of development, delivering and assessment due to its complexity. On the other hand, the absence of structured exercise intervention models for frail older people may be one of the obstacles to understanding the effectiveness of such programmes. Moreover, there is no structured guidance at the national level regarding the implementation of specific exercise interventions by a health or/and exercise specialist.

On the other hand, a physical exercise programme can be considered a complex intervention, since it is tailored to a specific population and setting and it is affected by several components regarding effectiveness and safety. The need to develop and validate well‐defined and replicable exercise protocols emerges to bridge the identified gaps. Thus, the exercise programme underwent the three stages proposed by Möhler et al. (2015): development, piloting and evaluation, having in mind that is it a good practice in clinical research.

As far as we are concerned, this is the first validation study following the revised guideline of Criteria for Reporting the Development and Evaluation of Complex Interventions in health care (CReDECI 2) by Möhler et al. (2015) to validate a physical exercise programme aimed at delaying the functional decline in frail older populations.

It should be noted that during the process of development of the physical exercise programme, the hypothesized effects of the intervention were not assessed regarding any of the outcomes of interest highlighted in the literature (e.g. gait, prevention of falls, cognition, quality of life and depressive symptoms). However, it has been found that, on a consensus basis, experts and potential programme users see this type of intervention as relevant and necessary in an institutional context.

Thus, a study protocol and a pilot study will be developed to test the effectiveness of the exercise programme on the promotion of independent living in frail older adults. If positive effects are observed, the programme can be considered for incorporation into the healthcare system and thereby contributes to the rehabilitation of older people who attend nursing homes.

### 4.1 Limitations

The main limitation of the study is that, although the revised guideline of Criteria for Reporting the Development and Evaluation of Complex Interventions in health care (CReDECI 2) by Möhler et al. (2015) was followed to validate a physical exercise programme aimed at delaying functional decline in frail older adults, this process does not guarantee the effectiveness of the intervention. Moreover, the process does not guarantee the absence of obstacles in the design, implementation or evaluation of a future larger‐scale study.

Other potential limitations regarding the delivery of the intervention or mode of action are related to the fact that during the pilot, the intervention was delivered by an external exercise specialist supported by the project. In the future, the exercise programme is supposed to be delivered by healthcare professionals supported by the app or e‐book and preferably, after attending a 2‐hr workshop. However, the intervention will be dependent on the institution’ strategy and vision, as well as on the motivation of professionals and participants. Another limitation is related to the specific and complex variables associated with gait assessment. The equipment is expensive and requires biomechanical methods background to collect, analyse and interpret data.

## 5 CONCLUSION

The CReDECI 2 process has the potential to help practitioners in developing and planning complex interventions, such as an exercise programme. An exercise programme aiming at managing the frailty condition was validated by healthcare and exercise professionals, nursing, physical therapy and exercise sciences students and the target population. The exercise programme includes several components that can be adjusted to the context and to the characteristics of the target population.

## CONFLICT OF INTEREST

No conflict of interest has been declared by the authors.

## AUTHOR CONTRIBUTIONS

RSR, JF, FR, NP, FCC, JA: Made substantial contributions to conception and design, or acquisition of data, or analysis and interpretation of data; RSR, JA: Involved in drafting the manuscript or revising it critically for important intellectual content; RSR, JA: Given final approval of the version to be published. Each author should have participated sufficiently in the work to take public responsibility for appropriate portions of the content; RSR, JF, FR, NP, FCC, JA: Agreed to be accountable for all aspects of the work in ensuring that questions related to the accuracy or integrity of any part of the work are appropriately investigated and resolved.

All authors have agreed on the final version of this paper and at least meet at least one of the four recommendations of the International Committee of Medical Journals Editors (ICMJE)—http://www.icmje.org/recommendations/. All authors did substantial contributions to the conception or design of the work; or the acquisition, analysis, or interpretation of data for the work. They also participate in drafting the work or revising it critically for important intellectual content.
